# Hepatitis B Virus Blood Screening: Need for Reappraisal of Blood Safety Measures?

**DOI:** 10.3389/fmed.2018.00029

**Published:** 2018-02-21

**Authors:** Daniel Candotti, Syria Laperche

**Affiliations:** ^1^Department of Blood-Transmitted Pathogens, National Transfusion Infectious Risk Reference Laboratory, National Institute of Blood Transfusion, Paris, France

**Keywords:** hepatitis B virus, transfusion, blood safety, nucleic acid testing, HBsAg, anti-HBc, residual risk

## Abstract

Over the past decades, the risk of HBV transfusion–transmission has been steadily reduced through the recruitment of volunteer donors, the selection of donors based on risk-behavior evaluation, the development of increasingly more sensitive hepatitis B antigen (HBsAg) assays, the use of hepatitis B core antibody (anti-HBc) screening in some low-endemic countries, and the recent implementation of HBV nucleic acid testing (NAT). Despite this accumulation of blood safety measures, the desirable zero risk goal has yet to be achieved. The residual risk of HBV transfusion–transmission appears associated with the preseroconversion window period and occult HBV infection characterized by the absence of detectable HBsAg and extremely low levels of HBV DNA. Infected donations tested false-negative with serology and/or NAT still persist and derived blood components were shown to transmit the virus, although rarely. Questions regarding the apparent redundancy of some safety measures prompted debates on how to reduce the cost of HBV blood screening. In particular, accumulating data strongly suggests that HBsAg testing may add little, if any HBV risk reduction value when HBV NAT and anti-HBc screening also apply. Absence or minimal acceptable infectious risk needs to be assessed before considering discontinuing HBsAg. Nevertheless, HBsAg remains essential in high-endemic settings where anti-HBc testing cannot be implemented without compromising blood availability. HBV screening strategy should be decided according to local epidemiology, estimate of the infectious risk, and resources.

## Introduction

Despite a vaccine and antiviral treatments being available, hepatitis B infection remains a global serious public health issue that affects more than two billion people worldwide. Hepatitis B virus belongs to the Hepadnaviridae family, which genome is a ~3.2-kb partially double-stranded circular DNA enclosed in an icosahedral capsid composed of HBV core (HBc) protein and an outer lipid envelope constituting the 30–42 nm in diameter viral particle. Three viral glycosylated surface proteins (large, middle, and small) embedded in the lipid envelop and are involved in virus binding of and entry into susceptible hepatocytes. During the viral life cycle, non-infectious subviral particles, designed HBV surface antigen (HBsAg), that lack the nucleocapsid and are composed of lipids and small surface proteins are produced in 1,000–10,000 excess compared with infectious virions (1). Due to its limited size, the HBV genome has a highly compact structure consisting in four overlapping reading frames for P, S, C, and X genes, which code for the reverse transcriptase/DNA polymerase, surface, core, and X proteins, respectively. The reverse transcription of a pre-genomic RNA intermediate during HBV replication contributes to a significant natural genetic diversity among viral strains. According to this genetic heterogeneity, HBV variants are classified currently into nine genotypes (A–I), some of them being further subdivided in subgenotypes (2). HBV genotypes and subgenotypes have different geographical distributions and are increasingly associated with differences in the natural history, clinical outcome of the infection, and detection. HBV chronic carriage prevalence varies according to geographical regions. Sub-Saharan Africa, South East Asia, China, and the Amazon Basin are highly endemic (≥8% HBsAg seroprevalence) or of higher intermediate endemicity (5–7.99%). Countries from the Mediterranean area, Eastern Europe, the Middle East, and North-West of South America are of lower intermediate endemicity (2–4.99%). Western and Northern Europe, North America, part of South America, India, and Australia have mostly low endemicity levels (<2%) (3).

HBV is transmitted through direct exposure to infected blood or organic fluids. The main routes of infection are sexual, vertical from an infected mother to her child during birth or shortly after, and parenteral including blood transfusion. Before 1970, approximately 6% of multi-transfused patients acquired HBV infection through transfusion. Over the past decades, the risk of HBV transfusion–transmission has been steadily reduced by the successive implementation of various safety measures that include donor selection based on risk-behavior evaluation, serological screening for HBsAg and antibodies against the core protein (anti-HBc), and nucleic acid testing (NAT) for HBV DNA. Nevertheless, hepatitis B remains a viral infection transmissible by transfusion with a residual risk varying according to HBV epidemiology, donor populations, and screening strategies (4). The HBV calculated residual risk estimate ranged between <1 and 1.4 per million donations in low-endemic countries and 16 and >100 in high-endemic countries (5–11). These estimates depend on the mathematical models used and are limited by the lack of recent published reports especially from sub-Saharan Africa. Nevertheless, the residual risk of HBV transfusion–transmission is associated mainly with blood donations tested negative for HBsAg and/or HBV DNA and collected during the early phase of primary infection or during the late stages of infection. Success or failure to intercept such potentially infectious donations may depend on the screening strategy and the performance of both serological and molecular assays used. Despite the existence of this residual risk, questions regarding the apparent redundancy of some of the safety measures implemented over the years (i.e., testing for two direct markers HBsAg and viral DNA) prompted debates on how to reduce the cost of HBV blood screening. However, it appears essential to consider carefully the potential negative impact on blood safety before considering removing any safety procedure, especially in high HBV prevalence settings.

The aim of this review is to examine the intrinsic limits and complementarity of HBV screening strategies of blood donations according to the epidemiologic situation.

## Blood Donor Selective Recruitment

In recent years, careful selection of blood donors became an essential and pragmatic element of blood safety management. In that respect, WHO actively promotes the recruitment of voluntary non-remunerated donors (VNRDs) (12). The generally high prevalence of bloodborne pathogens observed in paid donors supported this strategy. Blood safety is improved further by encouraging VNRDs to become regular donors who show considerably lower prevalence of viral markers (13). This policy was successfully implemented in most of high-income countries but might have negative consequences by excluding traditional family/replacement donors (FRDs) that constitute 4–100% of the blood supply in middle- and low-income countries (mainly in Latin America, Africa, and Central Asia), and therefore perpetuating blood shortage and increasing the cost of blood transfusion (14). Exclusion of FRDs relied mainly on the assumption that these donors could not be differentiated from unsafe paid donors. However, during the past few years, a wealth of evidence has been collected that showed no epidemiological and social difference between FRDs and first-time VNRDs (13, 15–17).

A second level of donor selection based on risk-behavior evaluation and at-risk exposure is used by most blood services worldwide to refuse high-risk individuals to donate blood temporarily or permanently. This procedure generally involves pre-donation risk assessment that requires first-time and regular donors to self-declare or self-complete a questionnaire every time before donation followed by a confidential interview with a medical counselor. However, the effectiveness of this donor self-deferral system strongly depends on donor education and accurate and truthful risk disclosure. Despite limited comprehensive data, the prevalence of overall non-compliance with transfusion-transmitted infection (TTI) risk-related deferral criteria was estimated between 1.65 and 13% in general donor populations, irrespective of blood screening results (18, 19). Studies exploring the rate of non-compliance reported substantially higher rates (~25%) among donors tested positive for viral infection(s) post-donation (20, 21). Recently, an overall 10% non-compliance rate was reported in HBV-infected blood donors from the Netherlands (21). Multiple and complex factors were found associated with non-compliance varying from deliberate (e.g., test seeking, social discomfort, disagreement with deferral criteria, and misunderstanding of the pre-donation screening purpose since donations are tested further) to genuine (e.g., misinterpretation of questions, failure of recall, and erroneous no-risk belief associated with temporally remote exposure) non-disclosures. Furthermore, a main risk factor associated with HBV infection in donors is to originate from an endemic region, and this cannot constitute selection criteria for obvious ethical and practical reasons. It would be unethical to consider this criterion for selection. Albeit the efficacy of donor risk-behavior selection is reflected by the significant lower prevalence of TTIs commonly reported among eligible donors compared with general populations, donor non-compliance may compromise transfusion safety and still needs to be minimized (22).

## Serum Alanine Aminotransferase Level Testing

Serum alanine aminotransferase (ALT) level testing was initially introduced in blood services as a surrogate marker for what was then called “non-A non-B” hepatitis and was later identified as hepatitis C. Elevated ALT level in an asymptomatic donor may constitute an unspecific marker for a wide range of active and potentially transmissible viral hepatitis infections (i.e., HBV, HAV, HCV, and HEV) (23). Therefore, exclusion of donors with elevated ALT is still used in several middle- and low-income countries, particularly where alternative molecular screening remains not affordable due to cost and technical constraints. However, ALT elevation could be mainly caused by various heterogeneous life style factors that are not related to viral infections and do not constitute a direct threat to blood safety. Unnecessary deferral of donors with elevated ALT might exacerbate the problem of blood shortage as debated in Japan and China where the ALT exclusion threshold was raised to 60 and 50 IU/L, respectively, in an attempt to mitigate the problem (24, 25). Following the implementation of effective serological and NAT for HCV and HBV, most of Western countries discontinued ALT routine donor screening as it was reported to have no significant added value in preventing HBV or HCV TTI (26, 27).

## HBsAg Testing

**Figure 1:**
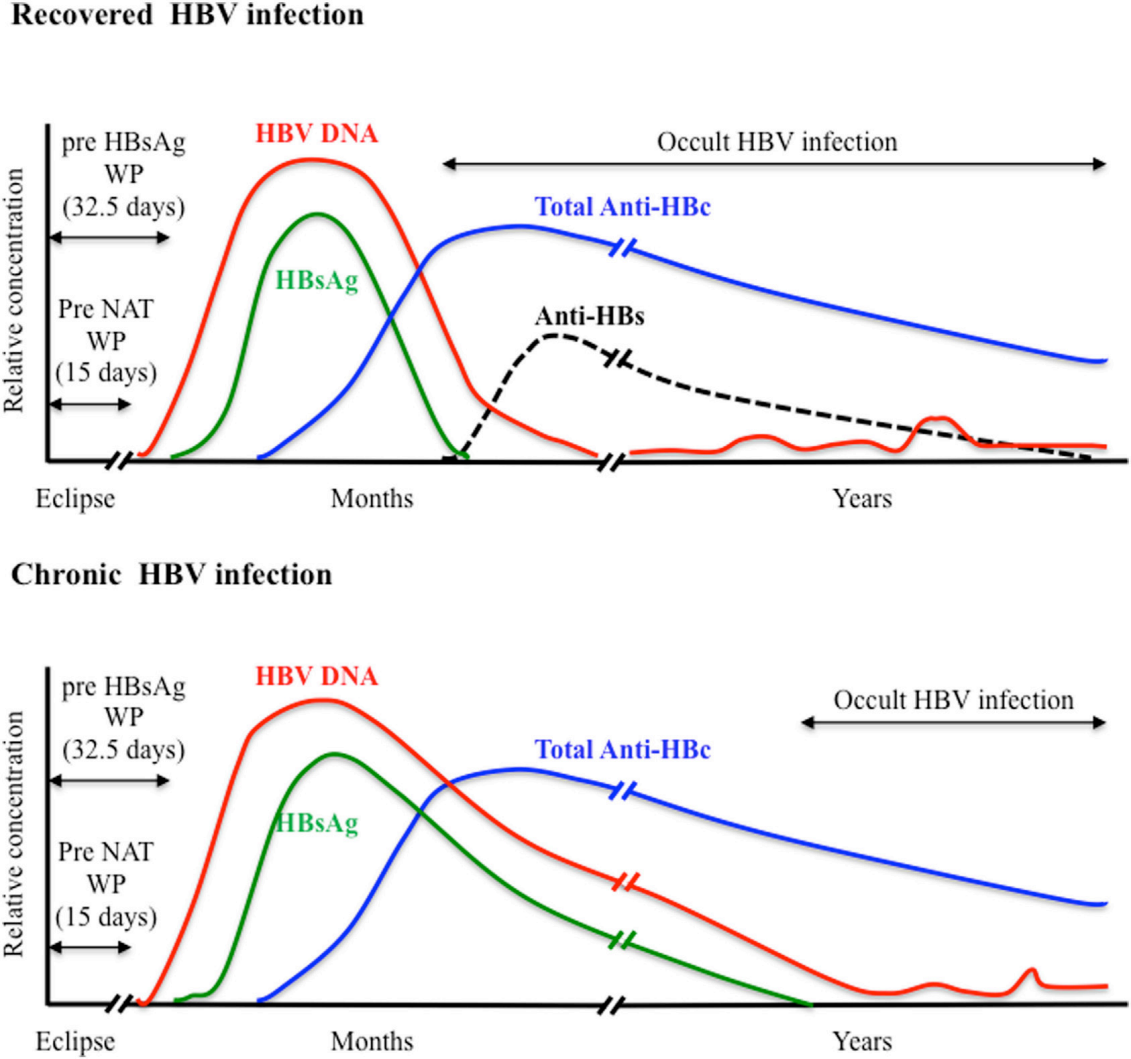
Viral plasma markers in recovered and chronic hepatitis B infection.

HBsAg is the first serological marker to appear during the course of HBV infection and remains the first line of HBV screening in blood donors. However, HBsAg screening required an optimal analytical sensitivity to limit the so-called “window period” (WP) phase, commonly defined as the time between infection and detection of the viral antigen, and to enhance the ability to detect the smallest amount of HBsAg during the asymptomatic late stage of chronic infection. Since the first assay available in 1970, the sensitivity and specificity of HBsAg testing has been steadily improving with the development of enzyme immunoassays (EIAs) including enzyme-linked immunoabsorbent assays that use chemoluminescence and polyclonal antibodies. A comparative evaluation of 70 HBsAg assays (51 EIAs and 19 rapid tests) from around the world indicated sensitivities ranging between 0.013 and 1 IU/mL for 84% of the EIAs tested (28). The pre-HBsAg WP was estimated to 32.5 days when using assays with <0.13 IU/mL sensitivity (Figure 1). Recently, an enhanced HBsAg chemiluminescent EIA (HBsAg-HQ) and an ultra-high sensitive HBsAg assay employing a semi-automated immune complex transfer chemiluminescence enzyme technique (ICT-CLEIA) were developed that showed 5 and 0.5 mIU/mL sensitivities, respectively (29, 30). These highly sensitive assays were reported to detect HBsAg before HBV DNA in few cases and to possibly reduce the WP to ~14 days (30, 31). However, they were developed mainly to monitor HBV reactivation in treated patients, and their suitability regarding blood donor screening has not been evaluated so far.

Although HBsAg EIAs proved to be effective in blood donor screening, they have many limitations in endemic low/middle-income countries that include high cost, need for sophisticated equipment and trained technicians, continuous supply of electricity, and long turnaround times. Despite showing reduced sensitivity ranging between 1.5 and >4 IU/mL compared with EIAs, rapid tests offer the advantage of low cost and rapid delivery of results and may constitute the only available HBV screening alternative in some resource-limited regions (28, 32–34).

Aside from the WP, HBsAg screening may fail to identify donors infected with HBV variants (35). Mutations within and outside the immunodominant regions of the S protein have been functionally associated with HBsAg structural changes that may lead to impaired detection by the current immunoassays (36, 37). These mutations may arise from escaping the host immune response during infection, vaccine, or HBV immunoglobulin treatment (36, 38). Because of the overlap of P and S ORFs, drug-selected changes in the reverse transcriptase/polymerase may also influence HBsAg detection (39). Recently, chronic HBV infection with antigen levels below the detection threshold of HBsAg assays was increasingly identified in donors and was defined as occult HBV infection (OBI) (40). Studies suggested that undetected HBsAg levels might be associated with mutations in the surface promoter impairing S gene expression or to mutations in the S protein and deletions in the pre-S1/S2 region that reduced HBsAg production and secretion from infected hepatocytes (41–44). In addition, the impact of HBV genotypes on the efficiency of HBsAg detection remains unclear. Albeit the most sensitive and commonly used HBsAg assays showed similar sensitivity in detecting all genotypes, some others had impaired sensitivity for genotypes D–F (28, 45). To overcome the risk of HBsAg false-negative results related to HBV variants, monoclonal antibodies were replaced by polyclonal antibodies against both “wild-type” and variant viruses. HBsAg assays using multiple monoclonal antibodies for capture together with a polyclonal conjugate for detection appear to be the most efficient in detecting a wide range of HBsAg epitopes. Another cause of HBsAg detection failure may be the formation of immune complexes in the presence of HBV surface antibodies (anti-HBs) (46). Furthermore, few studies described unusual cases of acute asymptomatic infections in blood donors detected by HBV NAT that, in contrast to overt acute HBV infection, never showed detectable HBsAg despite seroconverting to anti-HBc overtime and therefore were so-called acute primary OBI (47).

Generally, blood donor samples that initially reacted on a primary screening are retested either in duplicate with the same assay or with an alternative immunoassay. Despite a ≥99.5% specificity level estimated for the majority of HBsAg assays, repeat reactive samples not confirmed by further testing may represent either biological false-reactive or true positive with indeterminate testing results raising issues for donor management and unnecessary loss of blood components (28, 48). An Australian study reported similar HBsAg false-reactive rates of 0.02 and 0.03% in first-time and repeat donors, respectively (49). The causes of HBsAg false reactivity remain unclear but there were reports that HBV vaccination could result in a transient antigenemia in vaccinees (50). False reactivity appeared to be specific for an assay, mostly transient with ~85% of these donors found consistently negative at subsequent donations, and partially associated with low sample-to-cutoff (s/co) ratios (51). This predictive value of s/co ratios should be considered with caution as the s/co ratio distributions for false-reactive and confirmed-positive HBsAg results showed some overlap. Therefore, it is advisable that donors initially testing HBsAg repeat reactive are subject to serologic confirmation using a second immunoassay and a neutralization assay.

## ANTI-HBc Testing

Anti-HBc antibodies usually appear 6–12 weeks after infection are considered non-protective and remain detectable lifelong in immunocompetent subjects constituting the most sensitive marker for exposure to HBV irrespective of the current infection state (Figure 1). Anti-HBc may be the only serological marker of HBV infection at the end of a resolving infection when anti-HBs decline to undetectable levels or in OBI where HBsAg may be undetectable and HBV DNA only intermittently detectable (52–55). Recently, increasing evidence of HBV transmission by anti-HBc-reactive donors who repeatedly tested HBsAg and HBV individual donation (ID)-NAT negative with the most sensitive assays available has been reported (56–59).

Since it was first introduced in the late 1980s as a surrogate marker for non-A non-B hepatitis, anti-HBc screening for blood donors remains controversial. It is generally admitted that deferring anti-HBc reactive units would too severely affect blood supply and at a non-affordable cost in medium- and high-endemic areas where anti-HBc prevalence in blood donors ranges between 8 and >50% (i.e., Mediterranean area, East Asia, and sub-Saharan Africa). By contrast, the donor loss caused by universal anti-HBc screening was considered sustainable in some medium/low-endemic countries including Canada, France, Germany, Ireland, the Netherlands, Lebanon, and USA (60, 61). To limit potential donor loss associated with a ~5% anti-HBc prevalence, Japan implemented a complex screening algorithm that includes anti-HBs testing of anti-HBc only donations (56). Donations anti-HBc-reactive only that contain anti-HBs levels >100–500 IU/L are considered eligible for apheresis plasma donation for fractionation while red blood cells and platelets are discarded, and donations with low anti-HBc and anti-HBs levels are rejected. Plasmas from recovered anti-HBc-reactive individuals containing high levels of anti-HBs (e.g., >8,000 IU/L in France) still are needed to supply human hepatitis B immunoglobulin (HIBG) essential to prevent infection in immunosuppressed transplant patients and newborns from HBV-infected mothers. Setting of a minimum limit in anti-HBs titer (usually 500 IU/L) by plasma fractionators and/or national regulatory bodies and implementation of virus reduction procedures assure viral safety of products produced from anti-HBc positive plasmas (http://www.who.int/bloodproducts/publications/en/).

Blood products containing low levels of HBV DNA were found poorly infectious when transfused in the presence of anti-HBs (54). However, the protective level of anti-HBs remains a matter of debates as cases of HBV transfusion–transmission despite concomitant detectable anti-HBs were documented (56, 62, 63). Furthermore, the frequency of anti-HBs carriers among anti-HBc only donors may vary according to HBV epidemiology and vaccine coverage. Studies conducted in Europe, Japan, and North America reported that approximately 90% of anti-HBc-reactive donors carried also anti-HBs and 63–70% of them had titers >100 IU/L (56, 61, 64–66). By contrast, in Ghana, a country with high HBV endemicity, anti-HBs was detected in 24.5% of anti-HBc-reactive donors (67). Caution is required when comparing seroprevalences between studies due to differences in screening algorithms and methodology.

There are still limitations to anti-HBc screening even in low-endemic countries. Albeit recently improved, the specificity of anti-HBc testing is not optimal with reported false-reactivity rates of 16–75% according to assays and screening algorithms (60, 65, 66, 68–70). Recombinant/peptide antigen-based confirmatory assays being not available, secondary testing with an alternative EIA is needed to distinguish between true- and false-positivity and to confirm borderline reactive results that might be associated with low avidity or low titer of antibodies (65, 71). Additional testing for anti-HBs, anti-HBe, and/or HBeAg was considered to have confirmatory value for anti-HBc (56, 65, 66, 68). These complex confirmatory algorithms add economic and organizational constraints to blood services, but it is beneficial for the donor not to be permanently deferred due to false-positive outcome. Another limitation is that anti-HBc screening does not identify WP infections. In addition, simultaneous detection of HBV DNA and anti-HBs in the absence of detectable anti-HBc has been described. These cases were mostly associated with various degree of immunosuppression in patients, core regions deletion, and immunotolerance to HBc antigen in children born from HBeAg-positive mothers (45, 72, 73). However, rare anti-HBc negative/HBV DNA positive cases were also identified in immunocompetent blood donors irrespective of the presence of anti-HBs (53, 74, 75). The frequency of this unusual serological profile seems to vary according to the geographical origin of the donors and possibly vaccine coverage as it was detected in approximately 2 and 13% of OBI donors from Europe and Southeast Asia, respectively (53, 75).

## HBV NAT

Nucleic acid testing for HBV DNA was introduced initially in Austria, Germany, and Japan in the late 1900s. After 2004, its implementation for routine blood donation screening was extended worldwide when high-throughput commercial multiplexed NAT assays that included HBV DNA detection in addition to HIV and HCV RNAs were developed and licensed (76). The fully automated commercial multiplex (HBV/HCV/HIV) NAT assays mainly used in transfusion laboratories are the PCR-based cobas TaqScreen MPX version 1 or 2 assays (Roche Diagnostics), and the Procleix Ultrio or Ultrio Plus/Elite assays (Grifols Ltd.) that employ transcription-mediated amplification. The most recent cobas TaqScreen MPX v2 and Procleix Ultrio Plus assays showed specificity of 99.9% and similar 95% limit of detection (LOD) of 2–4 IU/mL for HBV DNA when applied to ID testing (77, 78). This high sensitivity allowed HBV NAT to reduce significantly the WP left by HBsAg testing to an estimated eclipse phase of ~15 days following infection (Figure 1) (79). In addition, HBV NAT uncovered a relatively large number of HBsAg-negative occult HBV infection (OBI) among blood donors who tested anti-HBc and/or anti-HBs positive (40). The majority of OBI donors are characterized by a viral load <50 IU/mL and, in some cases, the presence of a high amino acid variability within the S protein that might impair recognition by HBsAg assays (37, 53, 80). The sensitivity of HBV NAT not only depends on the efficiency of the amplification and detections methods used but also on the input plasma volume and the efficiency of the nucleic acid extraction (81). Moreover, the NAT analytical sensitivity may vary considerably between HBV genotypes and between strains of the same genotype, especially genotype D that is the most polymorphic types of HBV (82).

HBV NAT implementation may be limited by the considerable cost of high-throughput fully automated commercial platforms and reagents, especially in low- or medium-income countries of Africa, Asia, and South America. In high-income countries with usually low HBV prevalence, the clinical risk reduction benefit of NAT was associated with an extremely low cost-effectiveness (83). In addition to multiplexing, testing for viral genomes in plasma pools of various sizes was implemented to reduce the cost of NAT. However, there has been a constant progression toward screening smaller pools of six to eight plasmas and to ID. Indeed, the dilution factor introduced by the pooling process reduces the sensitivity of HBV NAT and its ability to detect the low levels of HBV DNA observed in the majority of OBI donors (9, 76, 78). Nevertheless, even ID-NAT may not be sensitive enough to detect potentially infectious blood products with extremely low levels of HBV DNA (56–58).

Discrepancies between serological and molecular testing and the increasing sensitivity of NAT assays make difficult to distinguish between true- and false-positive HBV DNA results. While the commercial multiplex cobas TaqScreen MPX v2 assay allows the simultaneous detection and direct identification of HBV, HCV, and HIV by using virus-specific probes labeled with different dyes, the cobas TaqScreen MPX v1 and Procleix Ultrio Plus assays indicate the presence of viral genomes with a single consensual signal that does not discriminate between these viruses. Therefore, three additional separate virus-specific discriminatory NAT assays are necessary to identify the virus in the originally reactive sample. Discriminatory assays do not fully qualify for confirmation since they are using the same technology and reagents as the initial screening assay. Furthermore, 0.09–0.29% of tested donations reactive in the initial multiplex assay might be non-reactive in the discriminatory assays and/or in the multiplex assay when repeated and were designed non-repeat-reactive (NRR) (55, 84–87). The reasons of these discrepancies remained unclear but probably reflect Poisson distribution statistics of HBV DNA levels around the assay’s LOD, especially in OBI donors, since multiplex and discriminatory assays showed no significant difference in sensitivity according to manufacturers (81, 85). Therefore, ID-NAT screened NRR donations are not released for transfusion in most countries, but donors may remain eligible to donate again as false-positive results cannot be totally excluded.

In the absence of serological investigations or detectable serological markers (i.e., WP), false-positive NAT results due to cross-contamination may be ruled out by retesting a clean sample from the initial plasma bag and by donor follow-up. However, caution should apply when considering the intermittently detectable HBV DNA levels observed in some OBI donors over time (53). NRR donations might be tested for anti-HBc to identify occult HBV carriers (88). NRR donations were reported more frequently reactive for anti-HBc than HBV DNA-negative donations (57 versus 7%, respectively) (85). However, this is not applicable in high-endemic countries such as China that showed an anti-HBc detection rate of 48% in HBV DNA non-reactive donations implicated in reactive minipools of 6 and 68% in ID-NAT NRR donations (86, 87). Alternatively, most ID-NAT users have adopted a serology-like algorithm to discriminate true from false initial reactive results. Multiple repeat tests are performed to identify NRR donations with low viral load using either the multiplex assay or a second independent commercial or in-house assay preferentially targeting a different region of the viral genome. This approach has its drawbacks as it is costly and NAT assays show different levels of sensitivity. Even the most sensitive assays may fail to detect extremely low levels of HBV DNA consistently (81). NAT sensitivity can be enhanced by several non-exclusive changes in the standard procedures aiming to increase the number of HBV DNA templates in the amplification reaction. This can be achieved by purifying viral DNA from larger volumes of plasma and/or by concentrating viral particles with high-speed centrifugation (84). Nevertheless, these approaches are not suitable for large-scale blood donation screening.

## HBV Screening Strategy: Are all Viral Markers of Value?

Blood donation screening for multiple HBV markers showed discrepant results. The frequency of these discrepancies is difficult to evaluate as they largely depend on the performance of the assays used. Nevertheless, a recent large-scale multiregional study using a comparable HBV screening algorithm showed that among 9,455 confirmed HBV-infected donors, 84.8% were consistently reactive for the three markers, 5.9% were anti-HBc and HBV ID-NAT reactive (OBI), and 2.65% were HBV DNA reactive only [WP (2.25%), primary OBI (0.13%), and anti-HBs only OBI (2.27%)] (89). In addition, 6.45% of donors were HBsAg and anti-HBc reactive but ID-NAT non-reactive. Previous studies reported absence of detectable HBV DNA in 2–20% of HBsAg reactive/anti-HBc reactive donors depending on the LOD of the molecular assays used (67, 76, 78, 82, 90). No confirmed HBV-infected donation testing HBsAg only has been identified so far.

In low-endemic affluent countries, the implementation of both HBsAg, anti-HBc, and HBV NAT provides the optimal level of blood safety by allowing detection of both the early phase of acute infection, persistent occult infection with potential transient detectable viremia, and genetic and/or antigenic viral variants. In addition, ID-NAT should be preferred, as it appeared more efficient in reducing the transmission risk by both WP and occult infections compared with MP-NAT (91). A residual risk would be left by the remaining early infection eclipse phase before HBV DNA becomes detectable. However, questions regarding the apparent redundancy of testing, especially for the two direct markers HBsAg and HBV DNA, prompted debates on how to reduce the cost of HBV blood screening without compromising blood safety. Accumulating data suggests that the apparently efficient combination of NAT and anti-HBc to detect both WP donations and low viremic chronic carriers precludes the need for HBsAg testing. There is increasing evidence that anti-HBc screening, if applied, would have interdicted infectious donations containing extremely low HBV DNA level undetectable with the most sensitive NAT (56–58). Despite being recommended by WHO and included in the European directive, the question of maintaining HBsAg testing might be raised but the absence of potential negative impact on blood safety needs to be assessed before considering discontinuing HBsAg. Therefore, the infectivity of such donations needs to be investigated. However, HBV infectivity studies are limited by the lack of physiologically reliable *in vitro* cell culture and susceptible animal models that generally require high doses of virus for infection (92). An alternative approach might be to isolate and amplify the viral genome present in HBsAg positive/HBV DNA negative donations and to use it in *in vitro* transfection experiments to study the virus replicative properties as a surrogate of infectivity. Dropping a screening test is highly challenging because it is politically sensitive and must not be perceived by the public as exposing recipients to higher risk. Solid scientific evidence about absence or minimal acceptable infectious risk should be provided to regulatory agencies and decision-makers who have the final decision.

In moderate- and high-endemic countries, anti-HBc testing cannot be implemented without compromising blood availability. Therefore, HBsAg testing in combination with NAT would be preferable when resource is available. Highly sensitive ID-NAT only might be considered, as it appears more efficient in detecting HBV chronic carriers than even enhanced sensitivity HBsAg assays. However, the existence of HBsAg reactive/HBV DNA non-reactive donations comforts maintaining HBsAg screening. In high-endemic countries with limited resource, HBV blood safety still relies essentially on HBsAg testing with inexpensive rapid tests as mentioned earlier. Pre-donation viral screening of blood donors using such rapid tests was shown effective and cost-effective, particularly in high-endemic areas (i.e., sub-Saharan Africa and China) where their use reduced wastage of collecting infected blood (93, 94). Additional testing of rapid test-negative donations with a different and more sensitive serological assay and/or expensive NAT still is needed to ensure an acceptable level of safety. The cost limitation of NAT may be addressed by developing in-house multiplex assays and/or by adapting assays using less expensive technologies that have been recently developed for monitoring viral infection at the point-of-care (93, 95, 96). In addition, quality assurance (QA) issues may persist in some resource-limited settings even with relatively simple serological assays such as HBsAg EIAs (33). Possible implementation of sophisticated but non-standardized in-house NAT assays may be prone to even bigger QA problems. Cheaper and well-validated commercial NAT assays may still be preferable to avoid false sense of biosecurity. However, the suitability of these new molecular methods for high-throughput blood screening remains to be evaluated. Discussions on the cost of NAT implementation must also take into account the multiplex format of the currently available systems that include HCV and HIV testing.

Decisions on screening strategy face the dilemma between cost-effectiveness and clinical benefit in terms of HBV TTI risk reduction. The HBV residual transmission risk depends essentially on the infectivity of the blood products from undetected HBV-infected donations. The minimum 50% infectious dose by transfusion was estimated between 20 and 200 IU (100–1,000 virions) in the absence of anti-HBs antibodies (54, 58). The HBV residual TTI risk may also vary according to the donation testing algorithms, the sensitivity of the serological and NAT assays used, and the HBV epidemiology. A recently developed mathematical model estimated this residual risk based on the probability distribution of the HBV DNA load in randomly selected OBI donors, the probability that a given DNA load remains undetected by NAT, and the probability that this DNA load causes infection in the recipient (4). According to this model, 3 and 14% of ID-NAT undetected OBI donations might cause infection by red blood cell concentrates and fresh-frozen plasmas, respectively. Another model based on lookback data reported similar 2–3% residual estimates of OBI transmission (58). When HBsAg and anti-HBc serology in combination with ID-NAT are used, the residual risk may be associated essentially with the remaining DNA-negative eclipse phase in early acute infection and the rare cases of anti-HBs only OBI with intermittent detectable DNA, albeit the infectivity of corresponding blood products is still unknown (75, 79).

Pathogen reduction technologies (PRTs) might represent an attractive strategy. Although PRTs are currently used to complement current testing, there are still limitations to overcome before considering it as a full alternative to testing. Indeed, PRTs were reported not 100% effective against infectious agents present in high loads (97). Efficacy of 2 to >5 log reduction in HBV infectivity has been reported using different PRTs (98). Therefore, the HBV infectious risk may be diminished but not eliminated since HBV viral loads ranging between undetectable to >10^9^ IU/mL are observed in blood donors (99, 100). In developed countries, PRTs are applied currently to fresh-frozen plasmas and platelet concentrates but remain unavailable for red cell concentrates. Some controversies also persist regarding their impact on the functional aspects of the treated components, albeit the clinical efficacy of treated products is generally satisfactory [see Ref. (98) for review]. Recently, the ability of pathogen reduction of whole blood to provide safer products at an affordable cost for low- and middle-income countries while retaining the ability to prepare functional components was raised (98, 101). The benefits of PRTs might be amplified in low-resource and high-risk countries due to the efficacy against different types of local bloodborne pathogens, including major TTIs (e.g., HBV, HCV, and HIV) and others widely endemic but yet unaddressed (e.g., malaria and bacterial infections). Few reports demonstrated that implementation of PRTs in resource-limited settings was feasible (98, 101). More studies are needed to assess the practical sustainability in terms of infrastructures, supplies, and cost-utility of PRTs implementation in settings where serology and NAT are already limited.

Finally, effective HBV vaccines have been available since the early 1980s, and vaccination has led to a 70–90% decrease in chronic HBV carrier rates in the countries where it has been implemented (102). Therefore, the extension of HBV vaccine coverage in both donor and recipient populations has the potential to reduce significantly the residual risk of HBV transfusion–transmission. However, 5–10% of healthy vaccinees failed to mount an adequate antibody response, vaccination alone failed to protect 10–30% of newborns from HBsAg/HBeAg-positive mothers, and occult HBV infection was frequently reported in individuals with protective anti-HBs levels. Suboptimal protection might be due to heterologous HBsAg (sub)genotypes or to the decline of anti-HBs level over time in vaccinees (63, 102). Nevertheless, a recently developed new generation of recombinant HBV vaccines that contain correctly folded HBsAg and additional neutralizing epitopes of the preS antigens was shown to be highly immunogenic, inducing faster and higher seroprotection rates against HBV compared with conventional vaccines. With optimal vaccines and vaccination coverage, eradication of HBV might be possible but that is another story.

## Author Contributions

DC and SL contributed equally to the conception and writing of the work and approved it for publication.

## Conflict of Interest Statement

The authors declare that the research was conducted in the absence of any commercial or financial relationships that could be construed as a potential conflict of interest.
